# Fever in the first month of life

**DOI:** 10.1186/1824-7288-41-S2-A8

**Published:** 2015-09-30

**Authors:** Paolo Biban, Simona Spada, Davide Silvagni, Silvia Perlini, Giovanna La Fauci, Chiara Ghizzi

**Affiliations:** 1 Department of Paediatrics, Paediatric Emergency Room, Azienda Ospedaliera Universitaria Integrata Verona, 37126 Verona, Italy

## Background

Fever is a common presenting sign in children and infants. However, the management of febrile neonates (< 30 days) could be particularly challenging, mainly due to the paucity of specific signs and symptoms to discriminate “simple, self-limited infections”, mostly of viral origin, from serious, life-threatening infections, mostly of bacterial aetiology. Despite several strategies and protocols have been proposed in the medical literature, management of fever do remain a complex issue in the neonatal patient.

## Contents

Evaluation of febrile newborns is primarily based on the clinical assessment, even though a combination of history, physical examination findings, and diagnostic screening tests is often required to exclude a serious illness reliably. Yet, in this high-risk group of patients, an adequate balance should be maintained, in order to appropriately identify and treat all sick newborns, while minimizing the risks associated with unnecessary invasive testing, hospitalization, and antibiotic treatment. Of note, practice guidelines for febrile neonates may differ substantially from centre to centre, in relation to execution and type of testing, intensity and level of treatment, and threshold for admission to the hospital.

According to several clinical practice guidelines, all febrile neonates should undergo a full sepsis evaluation and receive empirical antibiotics. Differently from older infants, in whom other risk factors must be taken into account, lumbar puncture is still widely recommended for any febrile newborn. Empiric antibiotic therapy usually consists in a combination of ampicillin and gentamicin, pending cultures results. In case of suspected herpes virus infection, i.v. acyclovir should be started immediately.

Finally, febrile newborns should be hospitalized or monitored in temporary observation units. Yet, consensus on the time of inpatient observation while awaiting culture results is lacking. In fact, despite a period of about 48-hour observation is generally accepted, recent data suggest that 24 hours could be adequate to detect most clinically significant bacteraemia [1]. Interestingly, an observation period beyond 24 hours would capture just one additional bacteremic infant for every 556 to 1235 febrile infants evaluated [1]. Indeed, by using this cutoff, the number of nights spent in the hospital for these infants and caregivers, as well as related costs, would be markedly reduced.

## Conclusions

Management of febrile newborns should include a full sepsis work-up, empiric antibiotics and strict monitoring in a hospital setting. However, recommended strategies are quite variable in the literature, and no protocol has been universally adopted as yet. A wide international consensus is still highly needed.

## References

[B1] Biondi EA Mischler M Jerardi KE Statile AM French J Evans R Lee V Chen C Asche C Ren J Shah SS Pediatric Research in Inpatient Settings (PRIS) Network: Blood culture time to positivity in febrile infants with bacteremia JAMA Pediatr 2014 81 844 849 25048522 10.1001/jamapediatrics.2014.895

